# Response of human leukocytes after stimulation with excreted-secreted toxocara canis larval antigens

**DOI:** 10.1186/1939-4551-8-S1-A47

**Published:** 2015-04-08

**Authors:** ANA Amor, Leonardo Santos, Márcia Silva, Eduardo Silva, Neuza Alcântara-Neves

**Affiliations:** 1 Universidade Federal Do Recôncavo Da Bahia, Brazil

## Background

Specific immunotherapy by administration of allergen extracts has been successfully used in asthma, allergic rhinitis and for venom. The identification of molecules which could be used as adjuvants in anti-allergy immunotherapeutic preparations is highly desirable. Anti-allergy immunotherapy has been associated with the induction of regulatory T cells. Helminths possibly downmodulate immune responses to airborne allergens, indirectly, through the stimulation of a regulatory network.

## Methods

In this study, it was investigated whether excreted-secreted *Toxocara canis* larval antigens (TES) (native) could elicit recall immune responses that could potentially inhibit a Th2 response, in nine allergic and ten non-allergic individuals' peripheral blood mononuclear cells (PBMC). PBMC were cultivated in vitro in the presence or absence of these extracts at 37 °C and 5% CO2 during 48 and 120 hours and their supernatants were evaluated for cytokine production (TGF-β, IL-10, IL-12, IFN-γ, IL-6, TNF-α, IL-5 IL13 and IL-17).

## Results

The antigen induced cytokine production in all PBMC preparations. Stimulation of the production of Treg cytokines (TGF-β and/or IL-10), accompanied or not by stimulation of cytokines production associated with the Th1 response (IL-12 and IFN-γ), but without stimulation of Th2 cytokines (IL-5 and IL-13) and IL-17, by antigen TES, was seen with 6 out of 10 allergic patients' PBMC.

## Conclusions

The results indicate that antigen TES induce Treg with or without Th1 immune responses. Searches of molecules, in this extract, which specifically induce this profile is suggested.

